# Distinct features of B cell receptors in neuromyelitis optica spectrum disorder among CNS inflammatory demyelinating diseases

**DOI:** 10.1186/s12974-023-02896-6

**Published:** 2023-10-04

**Authors:** Hyo Jae Kim, Jong-Eun Park, Wangyong Shin, Dayoung Seo, Seungmi Kim, Hyunji Kim, Jinsung Noh, Yonghee Lee, Hyunjin Kim, Young-Min Lim, Hyori Kim, Eun-Jae Lee

**Affiliations:** 1 Graduate School of Medical Science and Engineering, Korea Advanced Institute of Science and Technology, Daejeon, South Korea; 2 Department of Medicine, Asan Medical Institute of Convergence Science and Technology, University of Ulsan College of Medicine, Seoul, South Korea; 3 Bio-MAX Institute, Seoul National University, Seoul, South Korea; 4 Department of Electrical and Computer Engineering, Seoul National University, Seoul, South Korea; 5 Department of Neurology, Asan Medical Center, Ulsan University of Medicine, Seoul, South Korea; 6 Convergence Medicine Research Center, Asan Institute for Life Sciences, Asan Medical Center, Seoul, South Korea

**Keywords:** Inflammatory demyelinating disease of the CNS, Neuromyelitis optica spectrum disorder, Myelin oligodendrocyte glycoprotein antibody associated disease, B cell, B cell receptor

## Abstract

**Background:**

Neuromyelitis optica spectrum disorder (NMOSD) stands out among CNS inflammatory demyelinating diseases (CIDDs) due to its unique disease characteristics, including severe clinical attacks with extensive lesions and its association with systemic autoimmune diseases. We aimed to investigate whether characteristics of B cell receptors (BCRs) differ between NMOSD and other CIDDs using high-throughput sequencing.

**Methods:**

From a prospective cohort, we recruited patients with CIDDs and categorized them based on the presence and type of autoantibodies: NMOSD with anti-aquaporin-4 antibodies, myelin oligodendrocyte glycoprotein antibody-associated disease (MOGAD) with anti-myelin oligodendrocyte glycoprotein antibodies, double-seronegative demyelinating disease (DSN), and healthy controls (HCs). The BCR features, including isotype class, clonality, somatic hypermutation (SHM), and the third complementarity-determining region (CDR3) length, were analyzed and compared among the different disease groups.

**Results:**

Blood samples from 33 patients with CIDDs (13 NMOSD, 12 MOGAD, and 8 DSN) and 34 HCs were investigated for BCR sequencing. Patients with NMOSD tended to have more activated BCR features compare to the other disease groups. They showed a lower proportion of unswitched isotypes (IgM and IgD) and a higher proportion of switched isotypes (IgG), increased clonality of BCRs, higher rates of SHM, and shorter lengths of CDR3. Notably, advanced age was identified as a clinical factor associated with these activated BCR features, including increased levels of clonality and SHM rates in the NMOSD group. Conversely, no such clinical factors were found to be associated with activated BCR features in the other CIDD groups.

**Conclusions:**

NMOSD patients, among those with CIDDs, displayed the most pronounced B cell activation, characterized by higher levels of isotype class switching, clonality, SHM rates, and shorter CDR3 lengths. These findings suggest that B cell-mediated humoral immune responses and characteristics in NMOSD patients are distinct from those observed in the other CIDDs, including MOGAD. Age was identified as a clinical factor associated with BCR activation specifically in NMOSD, implying the significance of persistent B cell activation attributed to anti-aquaporin-4 antibodies, even in the absence of clinical relapses throughout an individual’s lifetime.

**Supplementary Information:**

The online version contains supplementary material available at 10.1186/s12974-023-02896-6.

## Introduction

CNS inflammatory demyelinating diseases (CIDDs) encompass a group of disorders, including multiple sclerosis (MS), neuromyelitis optica spectrum disorder (NMOSD), and myelin oligodendrocyte glycoprotein antibody-associated disease (MOGAD) [1]. These conditions are characterized by recurrent demyelinating attacks such as optic neuritis, transverse myelitis, and encephalitis throughout an individual’s lifetime [1]. While the identification of disease-specific autoantibodies has aided in their classification into distinct diseases [2, 3], B cells commonly play an important role in the development, progression, and relapse of CIDDs [4]. Consequently, B cell depletion therapies are currently employed in the treatment of CIDDs, with demonstrated efficacy [3].

NMOSD differentiates itself from other CIDDs due to its distinctive attributes, including severe clinical attacks with extensive lesions [5, 6], unique brain lesion patterns [7, 8], and association with systemic autoimmune diseases [9]. In particular, although NMOSD and MOGAD have a commonality in terms of the presence of autoantibodies targeting aquaporin-4 (AQP4) and myelin oligodendrocyte glycoprotein (MOG), respectively [10, 11], NMOSD exhibits different immunological characteristics from MOGAD. For instance, a higher proportion of plasmablasts is observed in NMOSD than MOGAD during the active phase, along with an increased intrathecal IgG index [12]. Pathological studies have also demonstrated differences in humoral responses between these two diseases [13]. These observations suggest the heterogeneity in the B cell-mediated humoral immune response, which could be associated with the clinical differences manifested in these conditions.

B cells recognize antigens through B cell receptors (BCRs) and function as effectors by secreting antibodies [14]. After encountering an antigen, activated B cells undergo somatic hypermutation (SHM) and affinity maturation [15]. Selective B cells with a high affinity for antigen proliferate and differentiate into memory B cells or antibody-secreting cells [16] and then undergo isotype class switching to function effectively [17]. The mRNA of BCR contains the aforementioned information [18], and recent developments in next-generation sequencing and bioinformatics have enabled the examination and comparison of BCR features in various conditions. Indeed, adaptive immune receptor sequencing, including T cell receptors as well as BCR, has been conducted in various systemic autoimmune and infectious disorders [19–21]. Analysis of these receptor features can also shed light on the distinct immune pathophysiology of CIDDs. However, research on BCR sequencing in CIDDs is at an early stage.

Considering differences in clinical characteristics and humoral responses between NMOSD and other CIDDs, we formulated a hypothesis that the intrinsic features of BCRs in NMOSD are distinct from those in other CIDDs. Therefore, our objective was to examine and compare the BCR features among patients with different CIDDs, with a specific focus on elucidating the differences between NMOSD and the other conditions.

## Materials and methods

### Participants

Since June 2018, we have been recruiting a prospective cohort of adult patients with CIDDs who visited the Department of Neurology at the Asan Medical Center (Seoul, Korea). In this study, we included patients from this cohort who visited the outpatient clinics in 2020 and used corticosteroids and/or azathioprine for maintenance treatment. Patients who had a history of using B cell depletion therapy, such as rituximab, were excluded. The participants were tested for anti-AQP4 and anti-MOG antibodies using a cell-based assay (Euroimmun, Lübeck, Germany) [22]. The presence of anti-MOG antibody was confirmed twice (> 1:40 titer) using a commercial fixed cell-based assay, as previously described in our publication [23]. We also conducted an additional assay for anti-MOG antibodies with a 1:100 dilution factor to determine whether the antibody presence is clear positive (a titer ≥ 1:100) or low positive (a titer ≥ 1:10 and < 1:100) [24]. Then, in accordance with the findings of the test, they were divided into the following groups: those with anti-AQP4 antibodies (NMOSD), those with anti-MOG antibodies (MOGAD), and those with DSN. We excluded patients from the DSN group who met McDonald’s criteria for MS [25]. As healthy controls (HCs), 34 volunteers without an infectious, immunological, or neurological disease were recruited. This study was approved by the Asan Medical Center Institutional Review Board (No. 2018-0653), and written informed consent was obtained from all participants.

### Clinical variables

The basic clinical information, including age, sex, comorbidities, and current medications, of all patients was obtained through the registry of the prospective cohort to which they belonged. A trained neurologist evaluated the Expanded Disability Status Scale (EDSS) score at the time of blood sampling. The following information was obtained regarding the disease course of the patient: (1) the total number of demyelinating attacks in the brain, spinal cord, and optic nerve; (2) disease duration from the onset date to the time of blood sampling; and (3) disease-free duration from the date of the last relapse to the time of blood sampling. In addition, information on serologic testing to evaluate other systemic autoimmune diseases was recorded, and, if available, follow-up AQP4 or MOG antibody testing results were gathered.

### Sampling and generation of heavy chain libraries

cDNA libraries were sampled and synthesized as previously described [26]. All blood samples were centrifuged, and the supernatant plasma was extracted. The remaining blood was combined with PBS and then transferred to a Ficoll solution to isolate peripheral blood mononuclear cells (PBMCs) via a gradient created by repeated centrifugation and washing. Immediately after collection, the PBMCs were cryopreserved in a cell freezing buffer (90% fetal bovine serum + 10% dimethyl sulfoxide) and then placed in a Frosty Freezing Container (Thermo Scientific, Waltham, MA, USA) for a week. After a week, the vials were transferred to a liquid nitrogen tank at − 192 °C. Total RNA was isolated from PBMCs using the TRIzol Plus RNA Purification Kit (Life Technologies). For library preparation, 1 µg of total RNA was employed as the input volume.

Then, reverse transcription was carried out with SuperScript IV reverse transcriptase (Life Technologies) and primers for seven immunoglobulin heavy chain isotypes containing unique molecular identifiers (UMIs) composed of 14 random nucleotides and partial Illumina adapters. First-strand cDNA was purified using AmPure XP beads (Beckman Coulter) at a 1:1.8 ratio, and second-strand cDNA was produced using KAPA HiFi HotStart DNA polymerase (Kappa Bioscience) and six immunoglobulin heavy chain variable region-specific primers. In a 1:1 ratio, double-stranded cDNA was purified using AmPure XP beads, following which it was amplified using KAPA HiFi HotStart DNA polymerase with double primers, including Illumina adapters and index. Using AmPure XP beads in a 1:1 ratio, the final next-generation sequencing (NGS) libraries were generated and submitted to quality control on TapeStation 2200 (Agilent Technologies). Libraries exhibiting a single peak of the correct sequence length were designated for NGS analysis with NovaSeq (Illumina).

### Preprocessing of raw data

The raw data were preprocessed following a previously described procedure [26]. Forward and reverse reads were assembled using PEAR v 0.9.10 [27]. The raw NGS reads were filtered on the basis that 95% had Phred scores of 20 or higher. The experiment’s primer regions were then retrieved from the reads. The UMI sequences were retrieved based on the location of the primer sequences, and the reads were grouped according to the UMI sequences. The reads within the same UMI clusters were aligned using Clustal Omega 1.2.4, a multiple sequence alignment tool [28]. Using the nucleotide frequency information of the alignment findings, the consensus sequences of the UMI clusters were extracted, and the read count of the sequences was redefined as the number of unique UMI sequences. In isotype annotation, the constant region was aligned with the constant gene database of the International Immunogenetics Information System [29]. The V/D/J genes and CDR1/2/3 regions were annotated by AssignGenes.py (ChangeO, Immcantation) and IgBLAST 1.19.0 [30, 31]. Following annotation, non-functional consensus sequences were eliminated using the same criteria as in prior studies: (1) sequence length less than 250 bp; (2) the existence of a stop codon or a frameshift in complete amino acid sequences; (3) failure to annotate one or more of the CDR1/2/3 regions; and (4) failure to annotate isotype [26].

### Features of B cell receptors

Clonal sequences were identified using DefineClones.py (ChangeO) with a nearest neighbor distance threshold calculated by distToNearest (Shazam v1.1.1) [31]. The clonal BCR is defined as follows: (1) identical V and J genes; (2) complementarity-determining region 3 (CDR3) has the same length, and (3) CDR3 nucleotide sequence differences are less than the Hamming distance threshold. The Hamming distance (the total number of character replacements required to make two strings equal) may be used to compare the difference between the CDR3 regions of the two BCRs. We selected the Hamming distance threshold calculated by distToNearest (Shazam v1.1.1) for each sample [31, 32]. For example, if the calculated threshold was 0.11, up to 11% of the nucleotide differences between the CDR3s of BCRs were identified as clones. Clonality was assessed by considering the proportion of BCR sequences forming a clone (excluding unique BCRs) within the total BCR sequence pool and the Shannon diversity [33]. SHM rate was computed based on the rate of nucleotide changes in the germline and the observed BCR sequences. CreateGermlines.py (ChangeO) was used to infer germline sequences for each clonal family, and observed mutations (Shazam) were used to compute the SHM rate for each BCR sequence [31].

### Statistical analysis

The isotype proportion, clonality, SHM rate, and length of CDR3 sequence were measured in this study. Independent *t*-tests, Mann–Whitney tests, or one-way analysis of variance (ANOVA) tests were used to compare numerical data, depending on the type of variable. For post hoc analysis involving multiple comparisons between groups, we applied the Tukey’s honestly significant difference (HSD) method for corrections. The correlation between BCR and clinical features was assessed using the Spearman’s rank correlation coefficient. All tests were two-sided, and a *p*-value of < 0.05 was considered statistically significant. All statistical analyses were performed using Python (version 3.9.1) or R (version 4.1.0).

## Results

We included 33 patients with CIDDs (13 NMOSD, 12 MOGAD, and 8 DSN) and 34 HCs (Table 1). Among MOGAD patients, 10 (83.3%) demonstrated clear positive results in the cell-based assay, while 2 (16.7%) revealed low positive results. Seven patients also underwent a live-cell assay during the disease course, and all were confirmed to have anti-MOG antibodies. The median (interquartile range) age of the patients from the CIDD group was 57.0 (44.0–62.5) years, and 22 (66.7%) were female. The patients from the HC group were younger (30.0 [27.0–32.8] years, *p* < 0.001) than those from the CIDD group and had a comparable number of female patients (19 [55.9%], *p* = 0.454). The patients from the disease groups showed no statistically significant differences in age (58.0 [49.0–63.0] in NMOSD, 59.0 [44.5–61.3] in MOGAD, and 46.0 [36.8–55.3] in DSN; *p* = 0.503). They also showed no significant differences in disease course, including the number of relapses, disease duration, or EDSS score. In terms of treatment, only patients using corticosteroids and/or azathioprine for maintenance treatment were included; the proportion of patients taking these medications was significantly lower in the DSN group than in the other groups (100.0% in NMOSD, 66.7% in MOGAD, and 25.0% in DSN; *p* = 0.001).

**Table 1:** Patient’s characteristics

	NMOSD (*n* = 13)	MOGAD (*n* = 12)	DSN (*n* = 8)	HC (*n* = 34)	*p*
Age, median (IQR)	58.0 (49.0–63.0)	59.0 (44.5–61.3)	46.0 (36.8–55.3)	30.0 (27.0–32.8)	** < 0.001**
Age at onset, median (IQR)	47.0 (43.0–51.5)	46.5 (19.5–57.0)	40.0 (23.5–46.5)	–	0.365
Female, *n* (%)	10 (76.9)	8 (66.7)	4 (50.0)	19 (55.9)	0.506
Number of attacks, median (IQR)	2.0 (1.0–3.0)	2.0 (1.5–3.0)	2.5 (1.3–3.0)	–	0.677
Optic neuritis	0.0 (0.0–1.0)	1.0 (0.0–3.0)	0.0 (0.0–0.8)	–	0.056
Brain	0.0 (0.0–1.0)	0.0 (0.0–0.8)	0.0 (0.0–0.0)	–	0.649
Myelitis	1.0 (1.0–2.5)	0.0 (0.0–1.0)	2.0 (0.3–3.0)	–	**0.005**
Disease duration, median (IQR)	7.7 (4.7–12.1)	5.9 (4.1–15.7)	8.7 (5.9–20.0)	–	0.696
Immune-modulating treatment^a^, *n* (%)	13 (100.0)	8 (66.7)	2 (25.0)	–	**0.001**
EDSS, median (IQR)	2.0 (2.0–3.8)	2.0 (1.1–4.3)	3.0 (1.5–4.6)	–	0.196

*p*-values < 0.05 were indicated in bold

DSN, double-seronegative; EDSS, Expanded Disability Status Scale; MOGAD, myelin oligodendrocyte glycoprotein associated disorder; NMOSD, neuromyelitis optica spectrum disorder

^a^Immune-modulating treatment including azathioprine and oral prednisolone

**Fig. 1:**
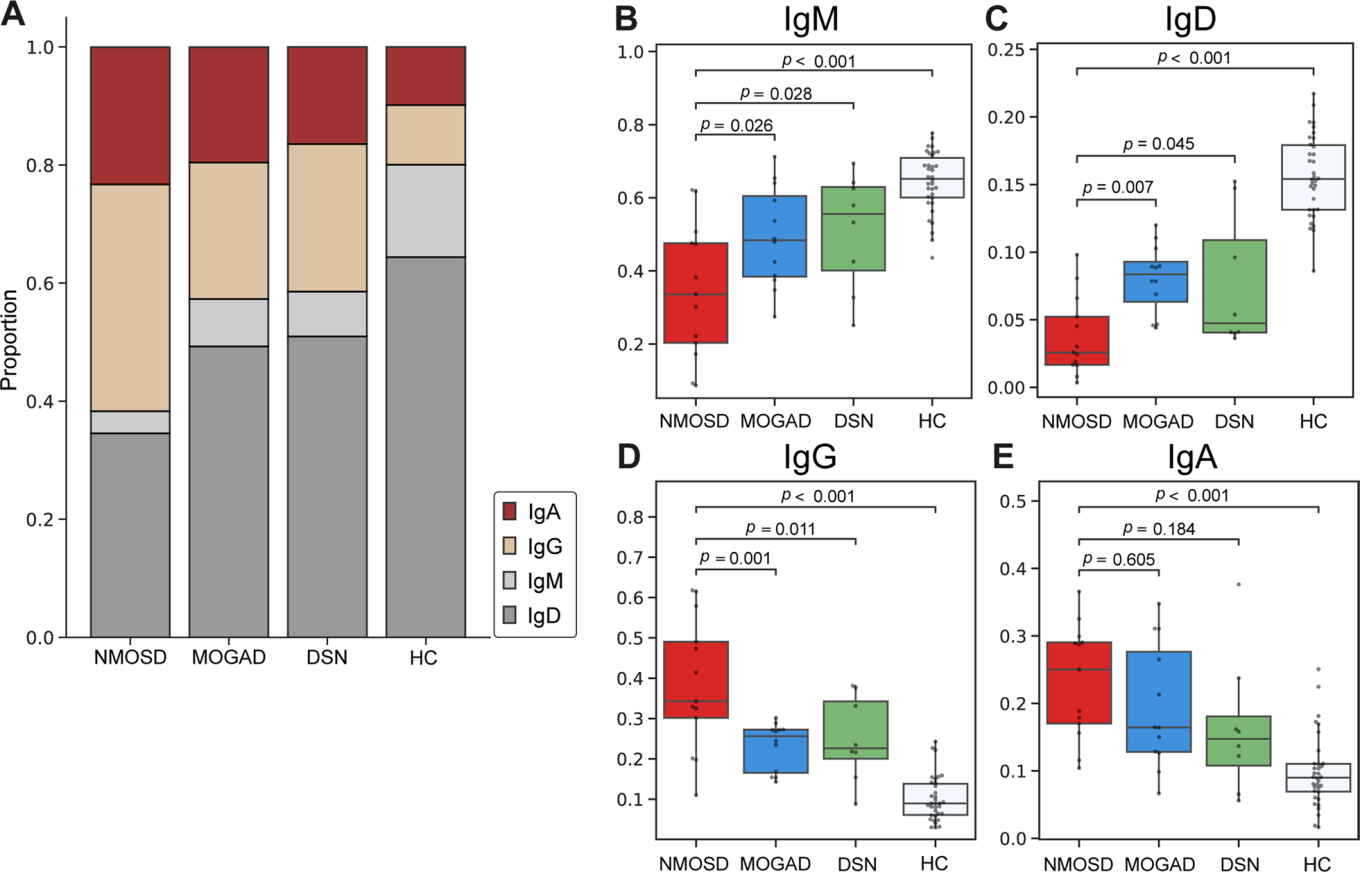
Comparison of isotype proportions among the different groups. **A** Bar plot illustrating the overall isotype proportion for each group. **B**–**E** Box plots for the comparison of IgM, IgD, IgG, and IgA isotype proportions between patients from the neuromyelitis optica spectrum disorder group and those from the other groups. The central line in the boxplot represents the median, and the error bars indicate standard deviation. *p*-values adjusted using Tukey’s HSD method

In BCR analyses, 8,996,152 read counts of preprocessed heavy chain sequences were obtained from 67 participants (NMOSD = 2,013,356; MOGAD = 1,992,573; DSN = 1,288,500; HC = 3,701,723). Comparison of the proportion of isotypes by group (Fig. 1A) showed that the proportion of BCR with unswitched isotypes (IgM and IgD) was greater and the proportion of switched isotypes (IgG and IgA) was lower in the patients from the HC group than in those from disease groups (NMOSD, MOGAD, and DSN). Comparison within disease groups (Fig. 1B–E) showed that the patients from the NMOSD group had a lower proportion of IgM and IgD than those from the two other disease groups (IgM: vs. MOGAD, *p* = 0.026; vs. DSN, *p* = 0.028; IgD: vs. MOGAD, *p* = 0.007; vs. DSN, *p* = 0.045). The proportion of BCR with IgG was greater in the patients from the NMOSD group than in those from the other disease groups (vs. MOGAD, *p* = 0.001; vs. DSN, *p* = 0.011); Among the subclasses of IgG, the proportions of IgG1 and IgG2 were higher in the NMOSD compared to the MOGAD group (*p* = 0.002 and *p* = 0.078, respectively) (Additional file 1: Fig. S1). Between the MOGAD and DSN groups, the proportion of BCR isotypes was comparable across the comparisons (Fig. 1B–E).

**Fig. 2:**
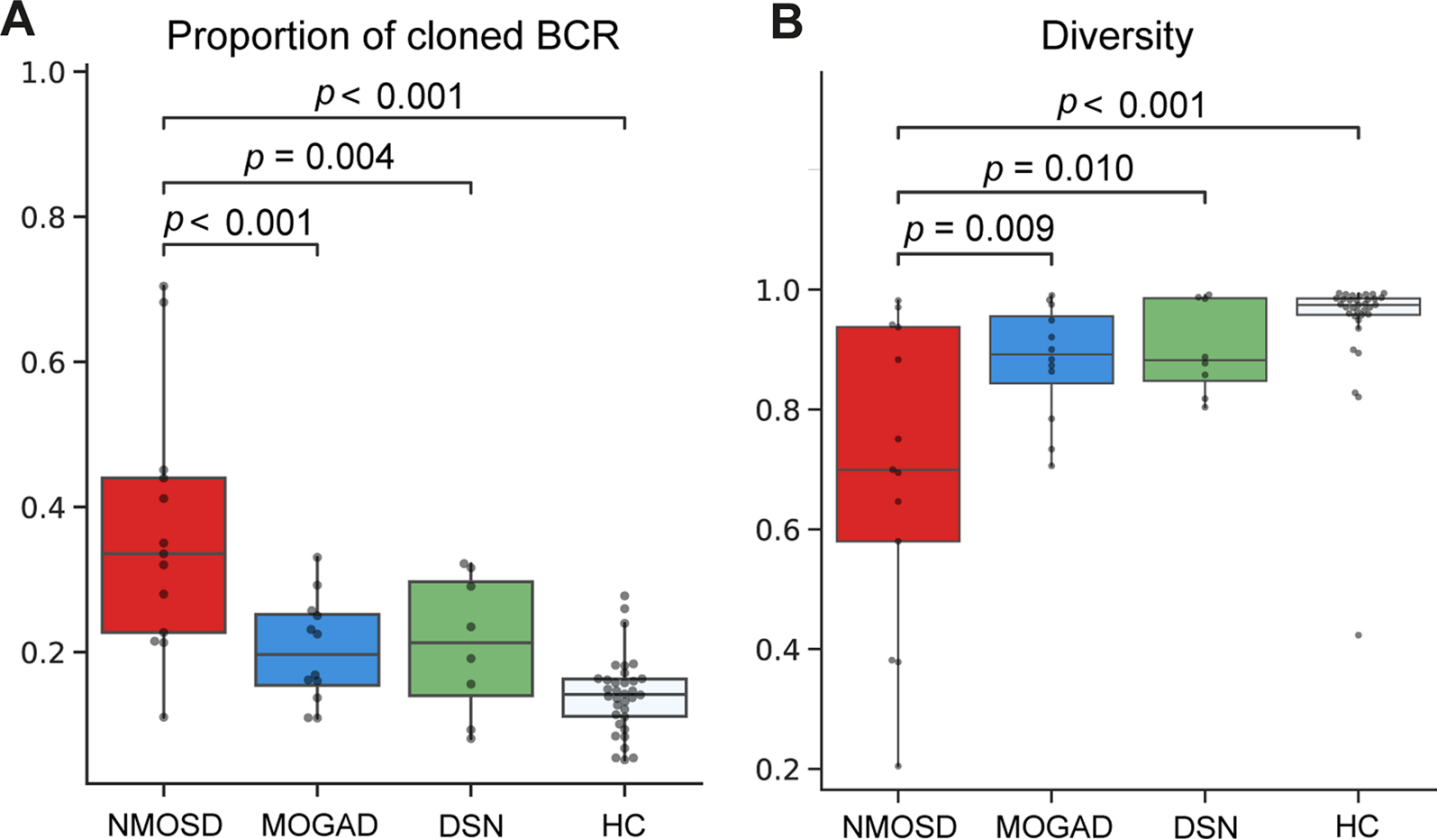
Comparison of clonality among the different groups. **A** Box plot showing the proportion of cloned B cell receptors (BCRs). The proportion of cloned BCRs was calculated as the ratio of BCR clones consisting of two or more to the total BCR count. **B** Diversity of BCRs among groups is presented in a box plot. The Shannon entropy method was used for calculating diversity. The central line in the boxplot represents the median, and the error bars indicate standard deviation. *p*-values adjusted using Tukey’s HSD method

The proportion of cloned BCR (clone size of two or more) was higher in the patients from the NMOSD group than in those from the other groups (36.4% in NMOSD vs. 20.3% in MOGAD vs. 21.0% in DSN vs. 14.1% in HC) (Fig. 2A). This increased clonality in the NMOSD group compared to the other groups was consistently observed when each isotype was compared separately (Additional file 2: Fig. S2). Diversity (Shannon entropy) was calculated to eliminate the effect of sequencing depth on clonality comparison (Fig. 2B), and results showed that the patients from the NMOSD group had the lowest diversity (highest clonality). The MOGAD and DSN groups exhibited a similar degree of proportion of cloned BCR and comparable diversity (Fig. 2A and B). Analysis of V gene usage frequency (Additional file 3: Fig. S3A) revealed that the usage of IGHV3-23 consistently increased in the patients from the CIDD groups compared with those in the HC group. Additionally, the frequencies of IGHV3–15, 3–21, 3–48, 3–7, and 3–74 increased, whereas those of IGHV1-69 and 4–39 decreased. In J gene usage, the frequency of IGHJ6 was lower in the patients from the NMOSD group than in those from the other groups (Additional file 3: Fig. S3B).

**Fig. 3:**
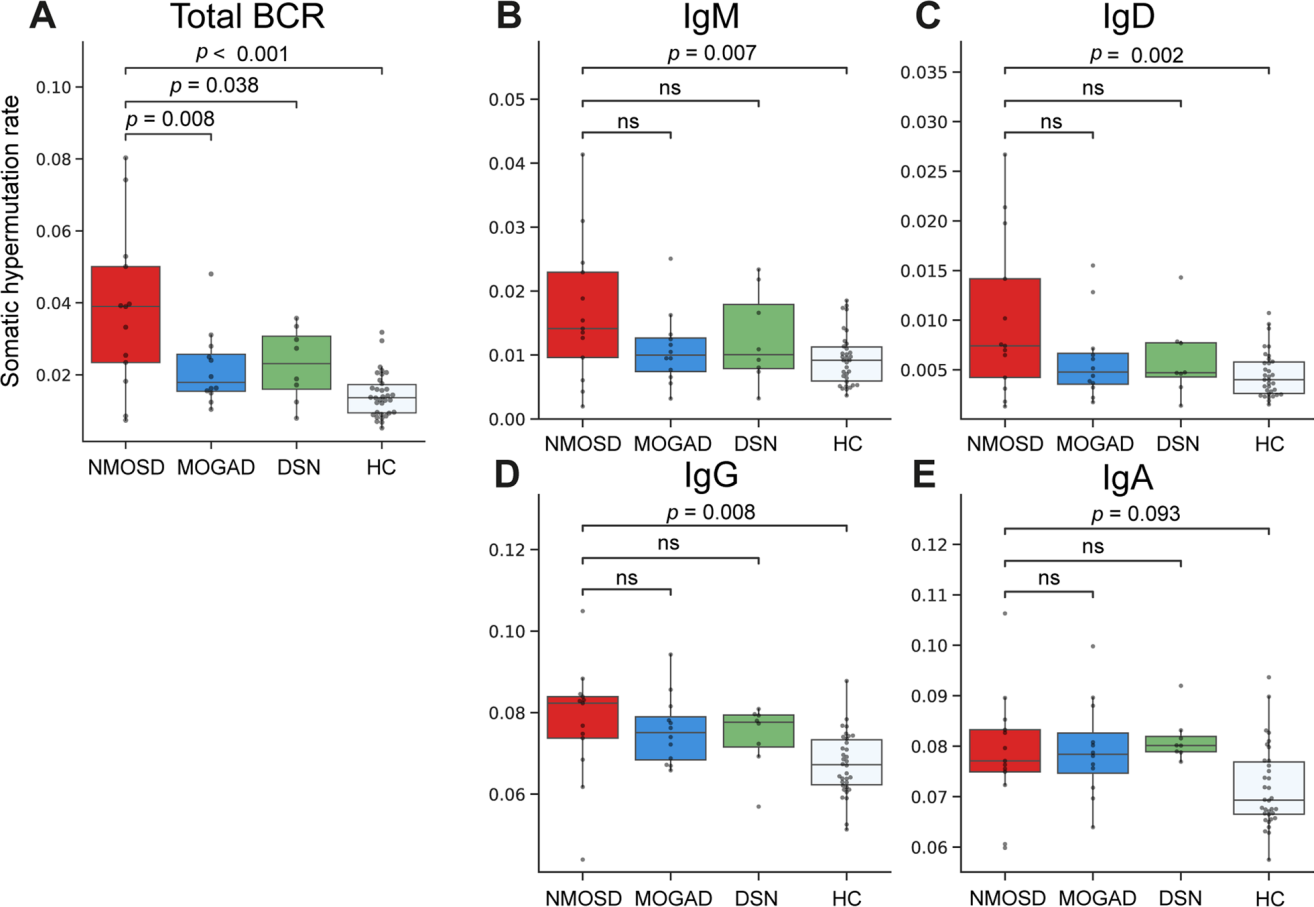
Comparison of somatic hypermutation rate among the different groups. **A** Box plot showing the overall somatic hypermutation rate for each group. **B**–**E** Box plots for the comparison of somatic hypermutation rate divided according to isotype (IgM, IgD, IgG, and IgA, respectively), between patients from the neuromyelitis optica spectrum disorder group and those from the other groups. The central line in the box plot represents the median, and the error bars indicate standard deviation. “ns” denotes no significance. *p*-values adjusted using Tukey’s HSD method

**Fig. 4:**
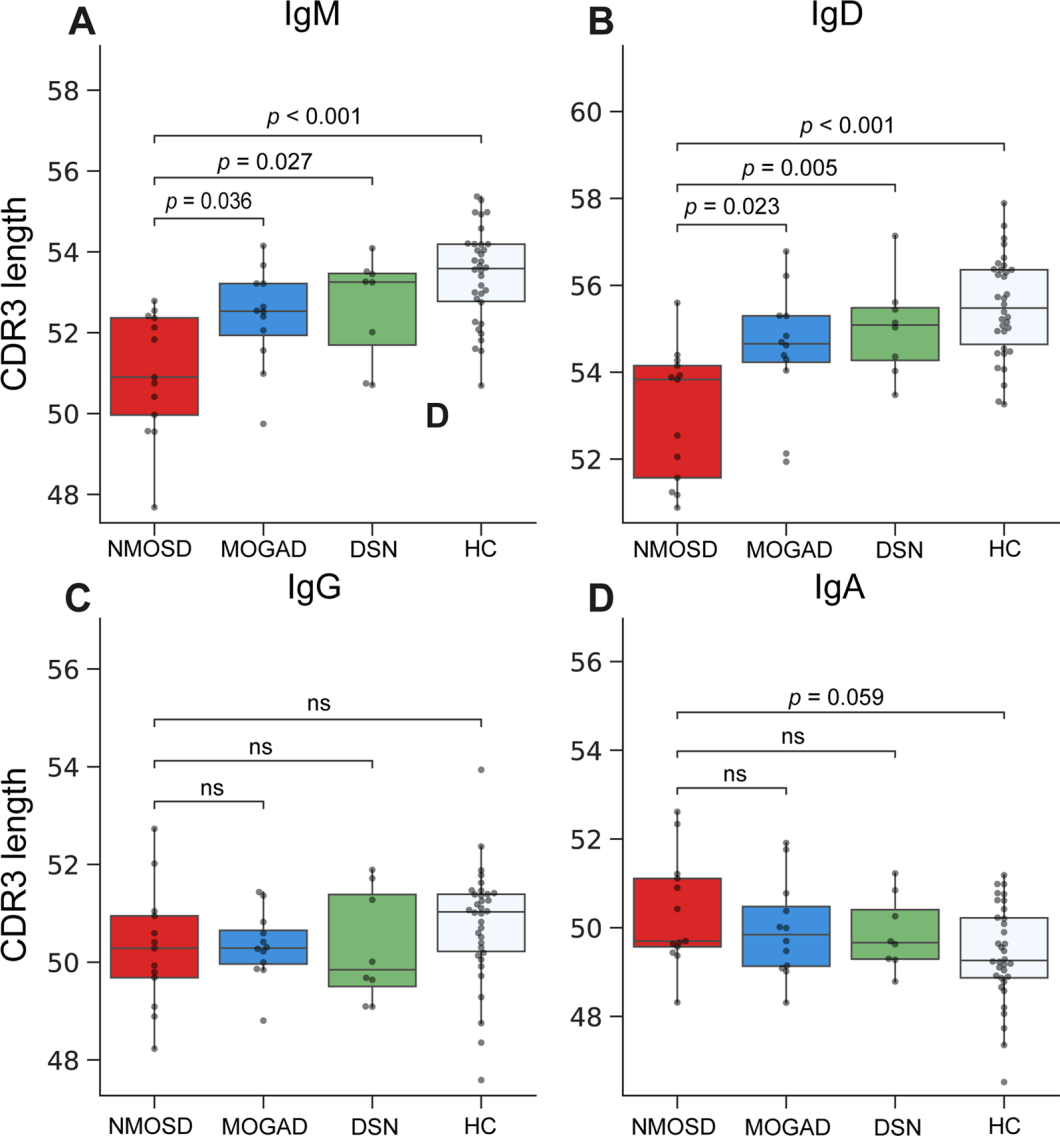
Comparison of CDR3 length among the different groups. **A**–**D** Box plots for the comparison of CDR3 length divided according to isotype (IgM, IgD, IgG, and IgA, respectively), between patients from the neuromyelitis optica spectrum disorder group and those from the other groups. The central line in the box plot represented the median, and the error bars indicate standard deviation. ‘ns’ denotes no significance. *p*-values adjusted using Tukey’s HSD method

As for mutation frequency, SHM rate was higher in the NMOSD group than in the other groups (total BCR: vs. MOGAD, *p* = 0.008; vs. DSN, *p* = 0.038; vs. HC, *p* < 0.001; Fig. 3A). In the comparison of SHM rate across the isotype (Fig. 3B–D), no specific isotype exhibited a notable increase in SHM rate. Meanwhile, SHM rates were comparable between the MOGAD and DSN groups (Fig. 3A). Comparison of CDR3 length between groups (Fig. 4) showed that the NMOSD group exhibited the shortest CDR3 length among BCRs with IgM or IgD isotypes. However, no significant difference in CDR3 length was found between groups for BCR with IgG or IgA isotypes. Furthermore, no significant differences were also identified between the MOGAD and DSN groups across all the isotypes.

**Fig. 5:**
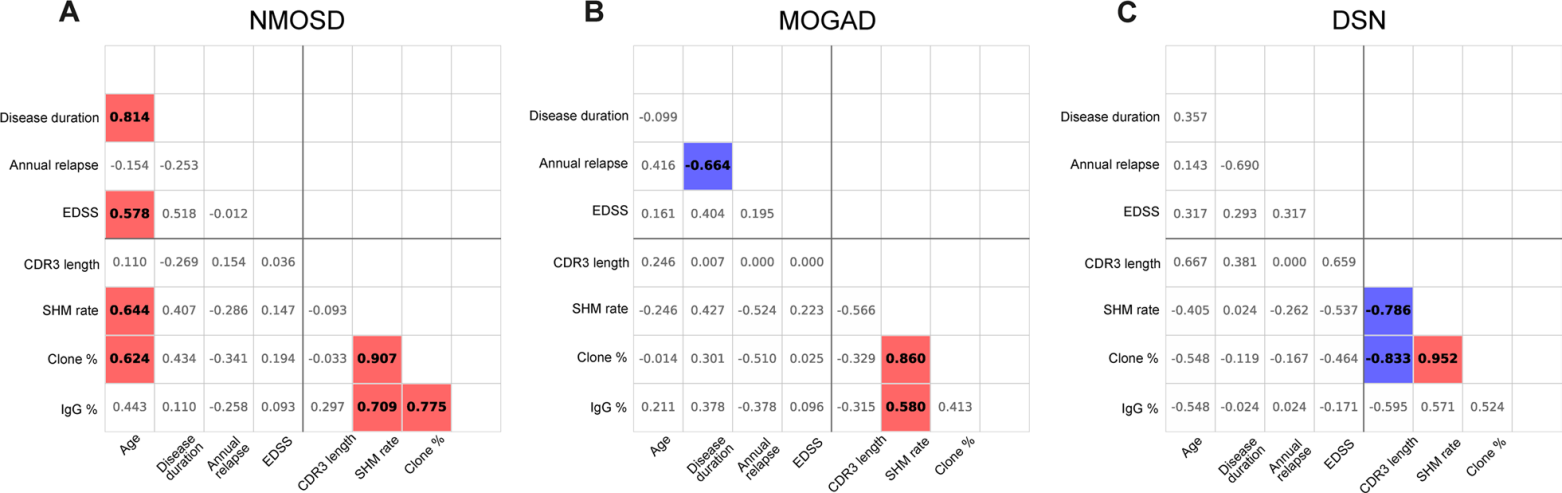
Relationship between B cell receptor characteristics and clinical features. This correlation matrix heatmap illustrates the relationships between the B cell receptor (BCR) characteristics and clinical features in neuromyelitis optica spectrum disorder (NMOSD) (**A**), myelin oligodendrocyte glycoprotein associated disease (MOGAD) (**B**), and double-seronegative disease (DSN) (**C**). Using the vertical and horizontal lines as references, the lower-left quadrant of the matrix represents the correlation between BCR and clinical features, whereas the lower-right quadrant shows the correlation among different BCR characteristics. Statistically significant coefficient R values (*p* < 0.05) are represented with a red background for positive R values and a blue background for negative R values

Finally, we investigated clinical factors including age, disease duration, and clinical severity that are associated with BCR features within each disease group. In the NMOSD group (Fig. 5A), age significantly correlated with higher clone proportion (*r* = 0.624, *p* = 0.023), and SHM rate (*r* = 0.644, *p* = 0.018). However, in the MOGAD and DNS groups (Fig. 5B and C), no significant relationship was found between BCR and clinical features. Correlation analysis among BCR features revealed that all three disease groups displayed a strong association between SHM rate and clone proportion. Of note, the SHM rate and clone proportion showed a significant negative correlation with the CDR3 length in the DSN group.

## Discussion

In this study, we evaluated BCR features in patients with various CIDDs and HCs. Our findings revealed that patients with NMOSD exhibit distinct BCR characteristics, demonstrating the most dramatic shift towards B cell activation. In addition, we identified age as a clinical factor associated with activated BCR features in NMOSD.

We focused on specific indicators derived from BCR sequencing data to evaluate B cell activation. These indicators included isotype switching, clonality, SHM rate, and CDR3 length. Assessing the proportion of isotypes switched to IgG or IgA provides information regarding the proportion of switched memory B cells or plasma cells, indicating the activation and class-switching of antigen-experienced B cells to different isotypes [34]. SHM is a crucial process in the germinal center where activated B cells undergo affinity maturation, enhancing the binding affinity of their BCRs [16, 35]. Therefore, the SHM rate can serve as an indicator of B cell activation. Clonality represents the humoral immune response, as clonal expansion is enhanced under conditions of continual stimulation with specific antigens, such as in autoimmune diseases. Finally, CDR3 length is shorter in memory B cells compared to naive B cells, and it becomes shorter with prolonged antigen exposure [36, 37], suggesting that shorter CDR3 lengths reflect B cell activation.

The notable finding of this study is the most heightened BCR activation in the patients with NMOSD. Specifically, the patients in the NMOSD group demonstrated significantly higher levels of B cell activation compared to those in the MOGAD group, despite both groups having respective pathogenic autoantibodies. The proportions of IgG, especially IgG1 and IgG2, in the patients from the NMOSD group were significantly larger than that in the patients from the MOGAD group, indicating that the factors driving isotype class switching, such as cytokines, may differ between the two groups. Additionally, the clonality and SHM rates in the patients from the NMOSD group were greater than those in the patients from the MOGAD group. The shorter CDR3 length of the BCR with an IgM isotype in NMOSD also implied a higher proportion of memory B cells and were associated with longer antigen exposure compared with those in the other disease groups. These findings suggest that the humoral immune response in patients with NMOSD is more pronounced than that in the patients with MOGAD. These observations align with recent research on the relapse patterns following B cell depletion therapy in patients with NMOSD and MOGAD [38]. Relapse in patients with NMOSD has been associated with the reactivation of B cells, whereas that in patients with MOGAD has been weakly correlated with the diminishing biological effect of rituximab [38]. In addition, approximately 20% of patients with NMOSD have intrathecal oligoclonal bands, but this is rarely the case with MOGAD [39, 40]. Therefore, the presence of expanded clones could be more prevalent in patients with NMOSD than in those with MOGAD. Even at the cellular level, in PBMCs, patients with NMOSD have greater proportions of plasmablasts and switched memory B cells than those with MOGAD [12, 41]. Additionally, patients with NMOSD exhibited higher levels of secreting IgG that correlated with plasmablasts, a finding not observed in MOGAD [42]. In the immunopathological study, perivascular deposits of activated complements and immunoglobulins, which are indicative of humoral immunity, were less prevalent in the brain lesions of patients with MOGAD than in those of patients with NMOSD [13].

The difference in humoral immune response between NMOSD and MOGAD may be ascribed to physical factors of self-antigen. AQP4 is not sequestered in the CNS and is also present in peripheral tissues [10], including the kidney, stomach, and airways, whereas MOG is restricted to the CNS. Furthermore, MOG is relatively rare in the CNS compared with AQP4 but concentrated in myelin [43]. Conversely, AQP4 is physically close to blood near the blood brain barrier. These anatomical differences may have contributed to a dissimilarity in the frequency with which the two self-antigens are encountered by immune cells, resulting in different humoral immune responses. Furthermore, it is known that defects in B cell tolerance are associated with the onset and progression of NMOSD [44]. Consequently, the immune system would react vigorously to self-antigens in NMOSD. The impaired B cell tolerance in patients with NMOSD may lead to a higher prevalence of systemic autoantibodies and increased B cell activation. In contrast, MOG protein is not expressed in the thymus and is sequestrated in the CNS [45], only incidentally exposed to peripheral immune surveillance, leading to the onset of the disease. As a result, the proportion of antibody-secreting cells in peripheral blood might be lower [12, 41], and the activity and degree of B cell activation could be relatively reduced. BCR characteristics and their clinical associations were exclusively observed in the patients from the NMOSD group. Older age was associated with activated BCR features, including the higher SHM rates and clonality. The prolonged exposure to self-antigens due to a longer disease duration may underlie these associations. However, the correlations between age and B cell features were more pronounced than those between disease duration and B cell features. These findings suggest that B cells remain persistently activated by AQP4 throughout individual’s lifetime, even before the onset of the disease or in the absence of clinical relapse events. This subclinical activation could be an important determinant for BCR characteristics, suggesting that persistent B cell activation may be the underlying mechanism for a modest yet ongoing subclinical degeneration in NMOSD [46].

B cell activation in the patients with DSN was comparable to that in the patients with MOGAD. Despite the absence of disease-specific antibodies, B cell-related pathology is recognized in MS, suggesting potential similar mechanisms in DSN [47, 48]. It is possible that B cell activation in DSN and MS is driven by CNS-derived antigens or by yet-to-be-identified disease-specific autoantibodies. Additionally, B cells play immunological roles beyond antibody production in CNS autoimmune diseases [49, 50], acting as antigen-presenting cells [51] and contributing to inflammation through cytokine secretion that stimulates T cells [52]. In these diverse immunologic processes, BCR stimulation and B cell activation are inducible.

This study has some limitations. First, this study sequenced the bulk heavy chain BCRs in PBMCs. Since the target condition was autoimmune disorders of the CNS, PBMCs may not adequately represent the status of intrathecal B cells. The majority of AQP4 antibodies are produced by extrathecal plasma cells. However, a recent study reported that a larger portion of MOG antibodies than AQP4 antibodies are produced by intrathecal plasmablasts migrated from peripheral blood [53]. Nonetheless, investigating the BCRs of PBMCs is essential. Immune cells are activated not only in the CNS, but also in the peripheral blood during neuroinflammation [54]. Therefore, the immune response of PBMCs may be a continuum rather than isolated from that of the CNS. Secondly, a fixed-cell based assay was used for the antibody test at the point of enrollment. Although the fixed-cell based assay is viable for the diagnosis of MOGAD [24], it does yield lower positive predictive values compared to live-cell assays [fixed vs. live-cell assay: 82.1 (64.2–92.2) vs. 100 (98.6–100) or 99.6 (97.9–100)] [55]. However, it should be noted that the majority of patients (> 80%) of patients had clear positive results, and all patients who subsequently underwent the live-cell assay demonstrated positive results. Additionally, the relatively younger age of the control group and the older age of the MOGAD group compared to typical cases are points to consider when interpreting the conclusions. Third, only patients in remission were included in the study. Thus, examination of the status of dynamic B cells in relation to disease course was limited. However, BCR characteristics developed throughout the acute period and did not disappear entirely. Memory B cells in peripheral blood circulate for several years to a lifetime and contain information about past antigens. In addition, the BCRs in patients in remission enable identification of the disease-specific properties of naive B cells. Future research should examine BCR features at multiple time points based on disease status.

In conclusion, among patients with CIDDs, patients with NMOSD exhibited the most significant B cell activation, as evidenced by increased levels of isotype class switching, clonality, SHM rates, and shorter CDR3 lengths. These findings suggest that B cell-mediated humoral immune responses in patients with NMOSD differ significantly from those observed in other CIDDs, including MOGAD. Notably, age was a clinical factor associated with BCR activation in NMOSD, suggesting the significance of persistent and subclinical B cell activation induced by anti-AQP4 antibodies throughout an individual’s lifetime in NMOSD.

### Supplementary Information

**Additional file 1: Figure S1.** Comparison of IgG subclass proportions for each group. **A–D** Box plots for the comparison of IgG1, IgG2, IgG3, and IgG4 isotype proportions between neuromyelitis optica spectrum disorder and the other groups. The central line in the boxplot represented the median, and the error bars indicated standard deviation. ‘ns’ denotes no significance. *p*-values adjusted using Tukey’s HSD method.

**Additional file 2: Figure S2.** Comparison of the proportion of cloned BCRs for each group, divided according to isotype. Box plots for the comparison of the proportion of cloned BCRs divided according to isotype (IgM, IgD, IgG1, IgG2, IgG3, and IgG4, respectively), between neuromyelitis optica spectrum disorder and the other groups. The central line in the box plot represented the median, and the error bars indicated standard deviation. ‘ns’ denotes no significance. *p*-values adjusted using Tukey’s HSD method.

**Additional file 3: Figure S3.** Comparison of V and J gene usage for each group. Bar plots representing the usage of V gene (**A**) and J gene (**B**) in the heavy chain. The Kruskal–Wallis test was used to assess statistical significance, with an asterisk *p-value of less than 0.05.

## Data Availability

Processed data used in this study are available on request from qualified investigators.

## Abbreviations

AQP4: Aquaporin-4
BCR: B cell receptor
CDR3: Complementarity-determining region 3
CIDD: Inflammatory demyelinating disease of central nervous system
CNS: Central nervous system
DSN: Double-seronegative disease
EDSS: Expanded Disability Status Scale
MS: Multiple sclerosis
MOGAD: Myelin oligodendrocyte glycoprotein antibody associated disease
NGS: Next-generation sequencing
NMOSD: Neuromyelitis optica spectrum disorder
PBMC: Peripheral blood mononuclear cell
SHM: Somatic hypermutation
UMI: Unique molecular identifier
