# Supplementary figures and images for: Distinct features of B cell receptors in neuromyelitis optica spectrum disorder among CNS inflammatory demyelinating diseases
Source: J Neuroinflammation. 2023 Oct 4;20:225. doi: 10.1186/s12974-023-02896-6 (PMC10548735; doi:10.1186/s12974-023-02896-6)

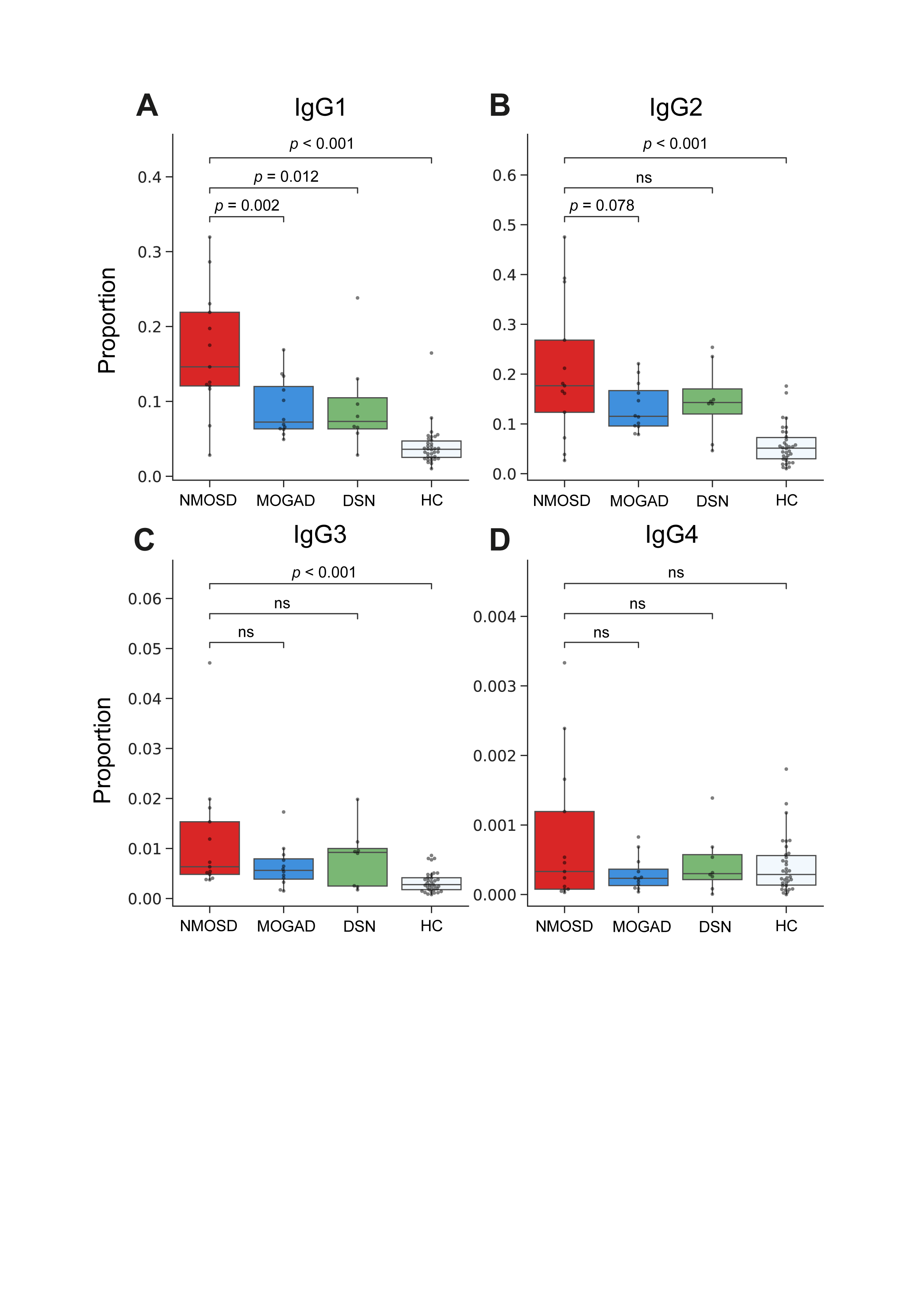

Supplement: Supplementary file 1 — Additional file 1: Figure S1. Comparison of IgG subclass proportions for each group. A–D Box plots for the comparison of IgG1, IgG2, IgG3, and IgG4 isotype proportions between neuromyelitis optica spectrum disorder and the other groups. The central line in the boxplot represented the median, and the error bars indicated standard deviation. ‘ns’ denotes no significance. p-values adjusted using Tukey’s HSD method. [file 12974_2023_2896_MOESM1_ESM.tif]

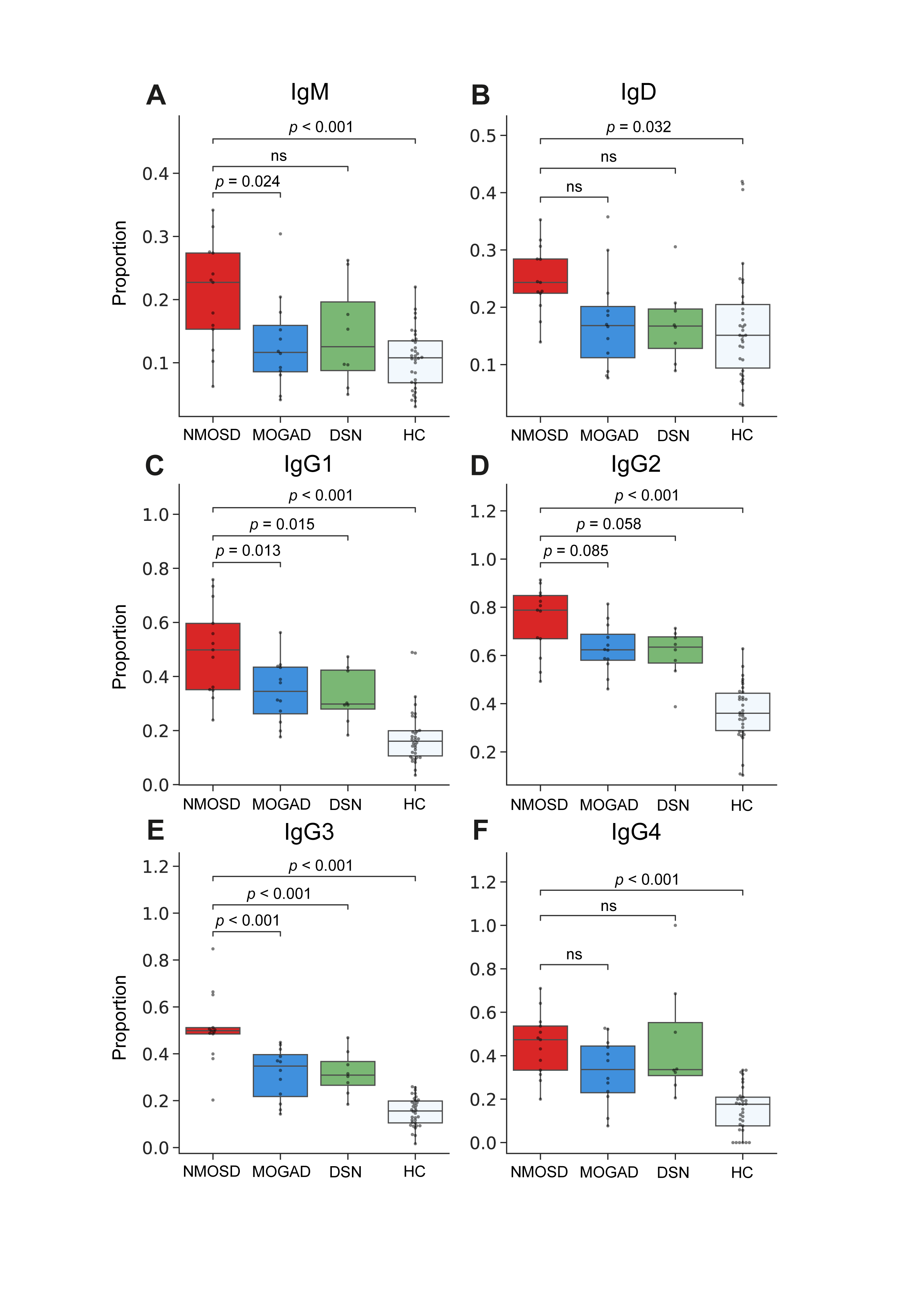

Supplement: Supplementary file 2 — Additional file 2: Figure S2. Comparison of the proportion of cloned BCRs for each group, divided according to isotype. Box plots for the comparison of the proportion of cloned BCRs divided according to isotype (IgM, IgD, IgG1, IgG2, IgG3, and IgG4, respectively), between neuromyelitis optica spectrum disorder and the other groups. The central line in the box plot represented the median, and the error bars indicated standard deviation. ‘ns’ denotes no significance. p-values adjusted using Tukey’s HSD method. [file 12974_2023_2896_MOESM2_ESM.tif]

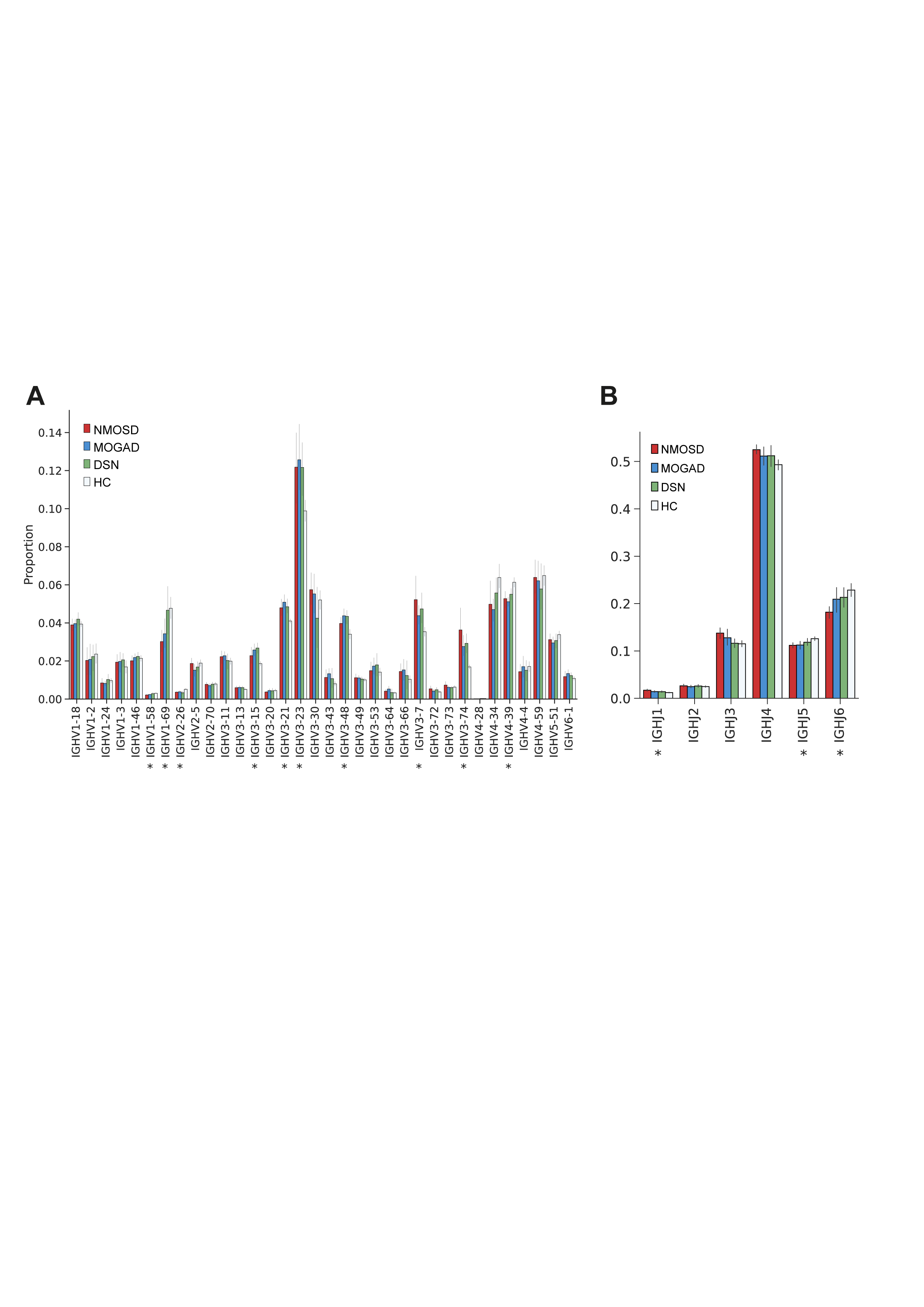

Supplement: Supplementary file 3 — Additional file 3: Figure S3. Comparison of V and J gene usage for each group. Bar plots representing the usage of V gene (A) and J gene (B) in the heavy chain. The Kruskal–Wallis test was used to assess statistical significance, with an asterisk *p-value of less than 0.05. [file 12974_2023_2896_MOESM3_ESM.tif]
